# PF-ViT: Parallel and Fast Vision Transformer for Offline Handwritten Chinese Character Recognition

**DOI:** 10.1155/2022/8255763

**Published:** 2022-09-28

**Authors:** Yongping Dan, Zongnan Zhu, Weishou Jin, Zhuo Li

**Affiliations:** School of Electronic Information, Zhongyuan University of Technology, Zhengzhou 450007, Henan, China

## Abstract

Recently, Vision Transformer (ViT) has been widely used in the field of image recognition. Unfortunately, the ViT model repeatedly stacks 12-layer encoders, resulting in a large number of model computations, many parameters, and slow training speed, making it difficult to deploy on mobile devices. In order to reduce the computational complexity of the model and improve the training speed, a parallel and fast Vision Transformer method for offline handwritten Chinese character recognition is proposed. The method adds parallel branches of the encoder module to the structure of the Vision Transformer model. Parallel modes include two-way parallel, four-way parallel, and seven-way parallel. The original picture is fed to the encoder module after flattening and linear embedding processing operations. The core step in the encoder is the multihead attention mechanism. Multihead self-attention can learn the interdependence between image sequence blocks. In addition, the use of data expansion strategies increases the diversity of data. In the two-way parallel experiment, when the model is 98.1% accurate on the dataset, the number of parameters and the number of FLOPs are 43.11 million and 4.32 G, respectively. Compared with the ViT model, whose parameters and FLOPs are 86 million and 16.8 G, respectively, the two-way parallel model has a 50.1% decrease in parameters and a 34.6% decrease in FLOPs. This method has been demonstrated to effectively reduce the computational complexity of the model while indirectly improving image recognition speed.

## 1. Introduction

Image Classification [1] and Chinese character recognition are important branches in the field of pattern recognition [2–4], which has attracted great attention and research in academic circles in recent decades. Chinese character recognition includes printed Chinese character recognition and handwritten Chinese character recognition (HCCR). Among them, handwritten Chinese character recognition can be subdivided into online and offline methods. Compared with the recognition of printed Chinese characters, the recognition of handwritten Chinese characters is more difficult [5] because people's different writing styles and habits for Chinese characters cause the randomness and complexity of handwritten fonts to increase. In addition, Chinese characters have the characteristics of a large number of categories (refer to the GB2312-80 standard; there are 6763 categories of commonly used Chinese characters) and many similar glyphs that are easy to be confused. It is constantly becoming a difficult point and a hot spot in research.

In daily life, like HCCR technology, handwritten numeral recognition technology is also widely used, such as for automatic sorting of postal codes, automatic recognition of check numbers, and correction of mathematics test papers. Handwritten numeral recognition has important research significance in the field of computer vision. It has attracted great attention from researchers. A new method for Arabic handwritten digit recognition based on the Boltzmann machine (RBM) and the CNN deep learning method is proposed [6]. The method has achieved a good recognition accuracy on the CMATERDB 3.3.1 Arabic handwritten digits dataset. In [7], a fusion-free method for multilingual handwritten digit recognition based on CNN is proposed, and this work uses CNN to solve the problem of multilingual digit recognition for the first time. The authors conducted extensive experiments on 8 numeric datasets in Indian and non-Indian scripts and finally achieved an accuracy of 96.23%. A new scheme for handwritten digit recognition based on mixed orthogonal polynomials is proposed in [8]. The scheme uses mixed orthogonal polynomials to extract gradient and smooth features and supports vector machines to identify and classify the extracted features of different numbers. The method achieves 99.32% and 100% recognition accuracy on the CMATERDB 3.3.1 and MNIST datasets, respectively. Compared with the research on the recognition of handwritten Chinese characters, the classification of handwritten digits has only 10 categories, with few categories and relatively simple strokes, so the recognition difficulty is not high. Therefore, this paper mainly studies the challenging handwritten Chinese character recognition problem. This work has great research value and significance.

Traditional HCCR mainly includes three steps: image preprocessing, feature extraction, and classification recognition. Commonly used methods of image preprocessing include sample normalization [9], shaping transformation [10], pseudo sample generation [11], etc., whose purpose is to enhance the useful features of the image and remove irrelevant noise, thereby extracting image features more conveniently [12]. Feature extraction includes two methods: structural features and statistical features. In the field of offline handwritten Chinese character recognition, currently commonly used and effective are the Gabor feature [13], Gradient feature [14], HOG feature [15], and other features methods. Commonly used classifiers include support vector machine (SVM) [16], linear discriminant analysis (LDA) [17], modified quadratic discriminant function (MQDF) [18], and learning vector quantity (LVQ) [19]. However, with the advancement of science and technology, the recognition accuracy and efficiency of traditional methods can no longer meet people's needs, and there is an urgent need to find some solutions to replace traditional Chinese character recognition methods.

In the 1980s, a convolutional neural network (CNN) was proposed, making it possible for researchers to apply CNN to Chinese character recognition systems. The effect of preprocessing and feature extraction in the traditional handwritten Chinese character recognition process is not effective, and using a CNN can automatically extract image features and process nonlinear relationships [20]. Feature extraction is one of the core problems in the field of computer vision, which mainly includes manual design and pure learning. The hand-crafted way of designing is the feature itself. According to the characteristics of human vision, the distinguishing features of the image are extracted, so the extracted features have specific meanings. These features can promote the processing and recognition of images. Meixner polynomials (MNPs) and their moments are considered important feature extraction tools. In order to solve the problem of instability of the coefficient values in the recursive algorithm in the case of high-order polynomials, Abdulhussain and Mahmmod [21] proposed a new recursive algorithm to compute the MNP coefficients of higher-order polynomials. The proposed algorithm derives an identity based on MNP, which reduces the number of recursions used and the number of MNP coefficients calculated. The Discrete Hahn polynomial (DHP) and its moments are one of the effective orthogonal moments, which are widely used in feature extraction. A practical method for computing the Hahn orthonormal basis has been proposed [22]. The authors apply this method to higher-order polynomials. The method consists of two recursive algorithms with adaptive thresholds to generate stable DHP coefficients.

In image recognition tasks, recognition accuracy is not only closely related to the model but also depends on image internal factors. Images of handwritten Chinese characters are affected by ambiguity, so it is difficult to distinguish the edge strokes of handwritten fonts, which has a certain impact on the accuracy of handwritten Chinese character recognition. A new fuzzy edge detector based on fuzzy divergence and fuzzy entropy minimization is proposed by Versaci and Morabito [23], and the correctness of the proposed method is verified in experiments. At the same time, the contrast of the image also has a huge impact on the recognition accuracy of the model. A fuzzy image preprocessor based on Euclidean space geometric calculation is proposed by [24], which improves the image contrast by correcting the histogram distribution of the original grayscale of the image through statistical geometric factors and entropy formulas. This method is characterized by a reduced computational load. Additionally, in [25], a fuzzy C-means clustering optimization method for leukemia detection based on morphological contour segmentation was introduced, including contrast enhancement to highlight nuclei, morphological contour segmentation, and fuzzy C-means to detect leukemia. This method is well suited for the identification and classification of leukocytes and leukemias. In conclusion, the above method is applied to the offline Chinese character recognition task, and the Chinese character recognition accuracy is improved to a certain extent.

The Transformer [26] is a typical deep learning model, which was first proposed as a sequence-to-sequence model for machine translation. This model mainly uses the self-attention mechanism [27, 28] to extract intrinsic features, which has a wide range of application potential in artificial intelligence applications. In 2020, the Vision Transformer (ViT) model [29] was proposed by Dosovitskiy et al., applying the attention mechanism to image recognition and classification tasks. This model can effectively extract the long-distance dependency information of the natural image itself. Also, it has reached or surpassed other methods on multiple image recognition classification benchmark datasets. However, it has the shortcomings of many model parameters and low efficiency in processing picture sequences.

The main contributions of our work are as follows:

- A parallel and fast ViT offline HCCR method is proposed. In this method, the encoder modules are arranged in parallel, and the parallel modes include two-way, four-way, and seven-way parallel. Among them, the best verification accuracy of the two-way parallel model reaches 98.6%. The experimental results demonstrate the effectiveness and correctness of the proposed method.
- The image is split into a fixed sequence of 16 × 16 patches and sent to the parallel encoder module. In the two-way parallel model, the minimum FLOPs are 4.32 G, and the parameter size is 43.11 million. The results show that parallel processing effectively reduces model computational complexity and FLOPs and indirectly speeds up image processing.

The rest of this article is structured as follows: Section 2 briefly reviews the related work.  Section 3 introduces the internal structure and related working principles of the ViT in detail.  Section 4 introduces the experimental procedures and experimental results in the dataset. The conclusion and future work are summarized in Section 5.

## 2. Related Work

### 2.1. Offline HCCR

With the advent of China's Industry 4.0, HCCR technology has been widely used, which is of great significance in the fields of handwritten Chinese character entry, automatic receipt recognition, and automatic scoring systems. After years of hard work and exploration, research scholars have achieved obvious breakthroughs and successes in the field of Chinese character recognition based on deep learning, especially CNN methods.

The multicolumn deep neural network (MCDNN) [30] obtained an accuracy of 95.78%, which opened the door to the application of the convolutional neural network model in the direction of HCCR. In 2014, the integrated model of alternating training relaxed convolutional neural networks (ATR-CNN) [31] was used by Wu et al. to improve the model and achieve an accuracy of 96.06%. A neural network model based on the backpropagation algorithm (BP) was proposed in the literature [32] to improve the recognition speed and accuracy of offline handwritten Chinese characters. The offline handwritten Chinese character recognition model HCCR-IncBN based on GoogLeNet was proposed in the literature [33]. The model obtained a recognition accuracy of 95.94%. An improved SqueezeNet model was proposed by Zhou et al. in the literature [34]. It retains the strategy of replacing the large convolution kernel with a small convolution kernel and uses a dynamic network surgery algorithm to ensure that important parameters that have been mistakenly deleted are respliced. The improved model has an accuracy rate of 96.03%. A summary table of offline HCCR-related work is shown in Table1.

### 2.2. Vision Transformer

After Transformer [26] was proposed, it has achieved very good performance in almost allnatural language processing tasks. Later, many researchers tried to establish a transformer model for visual tasks and achieved satisfactory results. These results indicate that the Transformer-based model has great potential in image recognition and classification [29].

A dual-branch Vision Transformer (CrossViT) for learning multiscale features was proposed in [35]. This method combines image blocks of different sizes to generate stronger image features. However, this method adds FLOPs and model parameters. A remote sensing image scene classification method based on the dual-branch structure of Vision Transformer and graph convolutional networks is proposed in [36], which forms a feature representation of long-distance dependency and spatial topological relationship fusion perception that can enhance the feature representation capability of the entire remote sensing scene image. A remote sensing scene classification method based on ViT is proposed in [1]. The compressed model obtained by the author after removing half of the multihead attention layer has an average classification accuracy of 97.90%, 94.27%, 95.30%, and 93.05% on the datasets Merced, AID, Optimal31, and NWPU. In [37], we first used ViT to classify breast ultrasound images and used different enhancement strategies. The final results showed that the ViT model and CNN have comparable efficiency in breast image classification and are even better than convolutional neural networks. A new architecture, the Convolutional Vision Transformer (CVT), was proposed in [38], which improves the performance and efficiency of ViT by introducing convolution in ViT. In addition, this method no longer requires positional embedding. The results show that the structure has achieved excellent performance while maintaining computational efficiency. A simple and effective method, reattention, was proposed in [39] to regenerate attention maps to increase the diversity of attention maps at different levels. This method can train a deeper ViT model through minor modifications to the existing ViT model and improve its performance. A summary table of Vision Transformer-related work is shown in Table 2.

After rapid development at home and abroad, ViT has also achieved good performance in computer vision tasks, such as detection [40], segmentation [41], tracking [42], image generation [43], enhancement [44], ancient text recognition [45], et al. In the future, the ViT will have a broad development prospect.

## 3. Methods

This paper proposes a parallel and fast ViT method for HCCR. The parallel methods are mainly divided into three types, namely, two-way parallel ViT, four-way parallel ViT, and seven-way parallel ViT. The core process of the two-way parallel ViT includes four main parts: image segmentation processing, linear embedding layer, position encoding, transformer encoder, and multilayer perceptron (MLP) classification processing. The core process of the four-way parallel and seven-way ViT is almost the same as the core procedure of the two-way parallel ViT. The difference is the number of parallel encoders and the number of repetitions of the encoder. The core process of the two-way parallel ViT is described in detail.

### 3.1. Two-Way Parallel Vision Transformer

**Figure 1:**
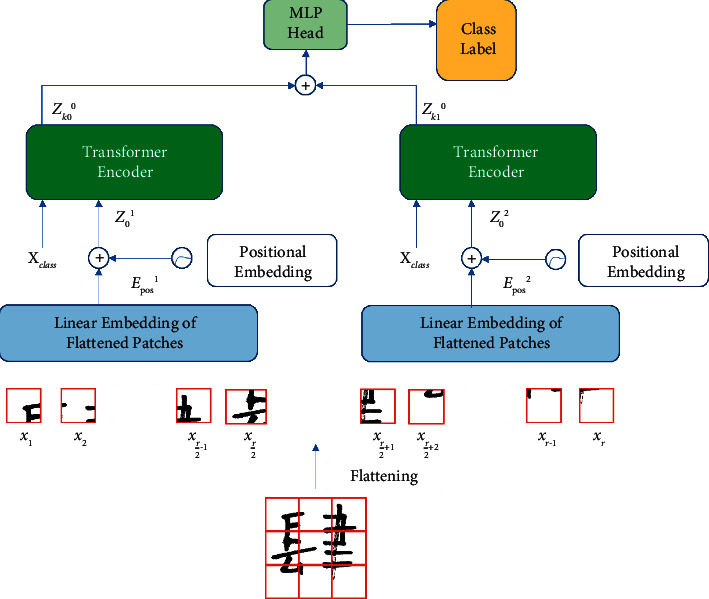
The overall architecture of the two-way parallel Vision Transformer.

The two-way parallel ViT retains the most original structural of the transformer design. Since the transformer model was proposed in 2017, it has been widely used in the field of machine translation and later achieved the most advanced performance in other natural language processing tasks and machine translation tasks. Strictly speaking, only the original transformer encoder module part is used in the two-way parallel ViT structure.  Figure 1 is the complete system architecture of two-way parallel ViT, including image block processing, a linear embedding layer, position coding, a transformer encoder, and MLP classification processing. When the picture sequence is fed to the encoder, the original long sequence needs to be divided into two short sequences and sent to the parallel encoder for processing, which can speed up the picture vector sequence, and finally, classify by mapping a series of image blocks to classification labels. The difference from the traditional CNN architecture is that the encoder part of the two-way parallel ViT uses the attention mechanism, which allows the model to focus on the information of different regions of the image and integrate the useful information of the entire image, which can improve the accuracy of image recognition.

#### 3.1.1. Image block Processing

The input of a traditional transformer is generally a sequence with labeled vectors, which is a two-dimensional matrix. For any picture *x*∈*R*^*H*×*W*×*C*^, where *H*, *W*, and *C,* respectively represent the height, width, and channel number of the picture in the dataset. First of all, the picture needs to be preprocessed and divided into small image blocks of the same size, which is also a very critical step. Each image block after segmentation is *x*_*i*_ ∈ *R*^*Q*×*Q*×*C*^, *(Q, Q),* which is the pixel of each block after segmentation. Then, each small image block is flattened, so that the original picture becomes a sequence of *r* image blocks, the sequence is (*x*_1_, *x*_2_,…, *x*_*r*_), where *r* *=* *HW*/*Q*^2^. Generally speaking, the size of each image block is generally 16 × 16 or 32 × 32. The smaller the size of each image block, the longer the vector sequence can be obtained.

#### 3.1.2. Linear Embedding Layer and Position Coding

The original image is divided into small image blocks, and each small image block becomes a one-dimensional vector after flattening and linear embedding and forms a long vector sequence. Then, both of these short sequences need to be processed by the linear embedding layer. The function of the linear embedding layer is to project the image block sequence into a D-dimensional vector through a learnable embedding matrix *E* for the linear embedding representation and to splice a learnable classification label *x*_class_ in front of the two short sequences. In addition, to keep the spatial arrangement of the image blocks consistent with the relative position of the original image, position information *E*_*pos*_^1^ and *E*_*pos*_^2^ need to be appended to the sequence representation. The position information here uses a simple one-dimensional position coding to retain the position information of the flattened image block. It has been verified in the literature [29] that the use of one-dimensional and two-dimensional position coding has a very weak effect on the recognition accuracy, but if the position coding is not used, the recognition accuracy will be reduced by about 3%. Finally, two embedded vector sequences *z*_0_^1^ and *z*_0_^2^ are obtained from a picture, as shown in formula (1) and (2).

(1) $$\begin{array}{c} z_{0}^{1}=\left[x_{class};x_{1}E;x_{2}E;\cdots ;x_{\left(r/2\right)}E\right]+E_{pos^{1}}, \end{array}$$

(2) $$\begin{array}{c} z_{0}^{2}=\left[x_{class};x_{\left(r/2\right)+1}E;x_{\left(r/2\right)+2}E;\cdots ;x_{r}E\right]+E_{pos^{2}}. \end{array}$$

#### 3.1.3. Transformer Encoder

**Figure 2:**
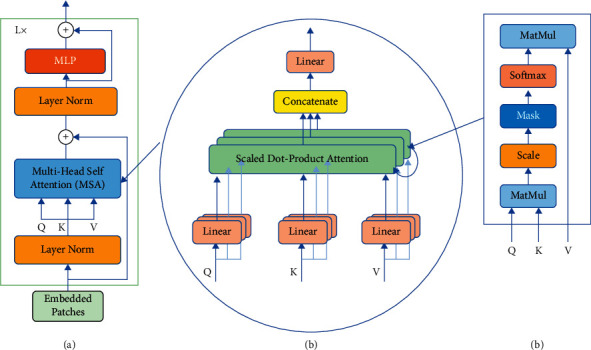
(a) Transformer encoder module; (b) multihead self-attention head; (c) self-attention head.

The two sequences *z*_0_^1^ and *z*_0_^2^ obtained after the linear embedding layer are sent to the transformer encoder, as illustrated in Figure 2(a). Each encoder is connected in series by multiple layers with the same internal structure. As can be seen from Figure 2, the encoder is mainly composed of two parts: the multihead self-attention (MHSA) mechanism and the multilayer perceptron (MLP), and the residual connection is also used. The multilayer perceptron is composed of two linear, fully connected layers, and the middle activation function uses the GELU function.

In addition, the two parts of the multihead attention mechanism and the MLP will go through a layer normalization (LayerNorm), as shown in formulas (3) and (4).

(3) $$\begin{array}{c} z_{k}^{\prime }=MHSA\left(LN\left(z_{k-1}\right)\right)+z_{k-1},k=1,\ldots ,K\left[1\right], \end{array}$$

(4) $$\begin{array}{c} z_{k}=MLP\left(LN\left(z_{k}^{\prime }\right)\right)+z_{k}^{\prime },k=1,\ldots ,K\left[1\right]. \end{array}$$

After the last layer of the encoder is processed, the first element of the sequence *z*_*k*0_^0^ and the sequence *z*_*k*1_^0^ are taken, respectively. They are superimposed and passed to an external classifier, after LayerNorm, to predict the class label and identify the picture category, as shown in formula (5) [29].

(5) $$\begin{array}{c} y=\text{LN}\left(z_{k0}^{0}+z_{k1}^{0}\right), \end{array}$$

The key component of the transformer is the MHSA structure of the encoder. This structure contains four layers, which are three parallel linear layers, a self-attention layer, a connection layer of multiple attention heads, and a final linear layer. As illustrated in Figure 2(b). The MHSA layer can determine the relative importance of a single image block embedded relative to other image blocks in the sequence. Attention can be represented by the attention weight, which is obtained by self-attention by calculating the dot product of *Q* (query), *K* (key), and *V* (value), and a weighted sum of all values of the sequence. Figure 2(c) shows the detailed process of calculation in the self-attention layer. After multiplying each element of the input sequence with the three learned matrices to generate *Q*, *K*, and *V*, the *Q* vector of each element is multiplied by the dot product of the *K* vectors of other elements. Then divide by the square root of the dimension of *K* and send it to the softmax function. Finally, multiply the output value of softmax by the *V* vector of the element to obtain an image block with a higher degree of attention. The calculation process is shown in formula (6) [39].

(6) $$\begin{array}{c} Attention\left(Q,K,V\right)=softmax\left(\frac{QK^{T}}{\sqrt{d_{k}}}\right)V. \end{array}$$

MHSA first takes a linear transformation of *Q*, *K*, and *V*, and inputs it to the scaled dot product attention, and then does it *h* times instead of just once. *h* is the set number of multiple heads. Finally, the *h* times scaled dot product attention results are spliced together and linearly transformed to obtain the final result. Formulas (7) and (8) express the calculation process [36].

(7) $$\begin{array}{c} head_{i}=Attention\left(QW_{i}^{Q},KW_{i}^{K},VW_{i}^{V}\right), \end{array}$$

(8) $$\begin{array}{c} MultiHead\left(Q,K,V\right)=Concat\left(head_{1},\ldots ,head_{h}\right)W^{O}. \end{array}$$

#### 3.1.4. Multilayer Perceptron classification Processing

**Figure 3:**
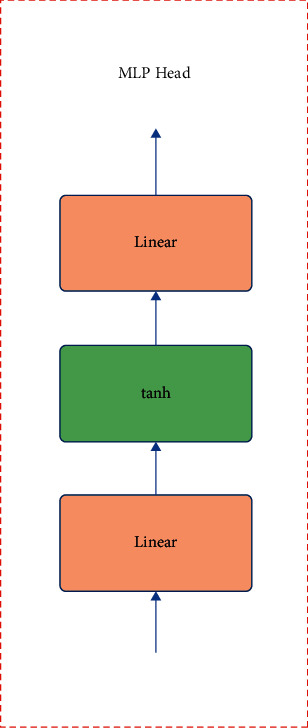
Multilayer perceptron head structure.

MLP includes an input layer, a hidden layer, and an output layer. There can be multiple hidden layers or one intermediate. The simplest one has only one hidden layer. At this time, the MLP has only a simple three-layer structure. Moreover, the layers are fully connected, as shown in Figure 3. The structure of MLP classification differs for different datasets. Generally speaking, when training a large dataset such as ImageNet21K, the MLP is composed of two linear layers and a tan*h* activation function. If it is applied to a relatively small dataset, such as ImageNet1k or its own dataset, only one linear layer is sufficient.

### 3.2. Data Expansion Strategy

For large-scale network models, it is often necessary to train a large amount of data. A dataset with a small amount of data can no longer meet the training needs. Therefore, a simple and effective strategy is needed to increase the number and diversity of training samples in the dataset. The commonly used strategy is data expansion.

**Figure 4:**
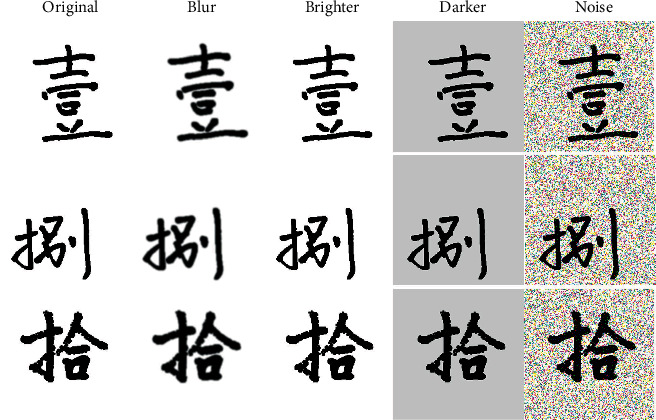
An example of applying data augmentation to a dataset.

Data expansion aims to generate additional training data based on existing training data samples. Basic data expansion methods include simple geometric transformation types such as flipping, deformation scaling, cropping, and color transformations such as adding noise, color contrast transformation, and blurring. This article mainly uses the methods of blurring, adjusting the brightness and darkness of the image, and adding Gaussian noise to expand the dataset. This not only improves the generalization ability and robustness of the model but also effectively overcomes the overfitting problem in the training process.  Figure 4 is an example of applying data augmentation to some samples in the dataset.

## 4. Experiments

### 4.1. Dataset

In this experiment, the HCCR dataset was made by us. The dataset is named DHWDB. The characteristics of the dataset are shown in Table 3. Finally, it contains 36210 images with 16 classes. The picture size is 224 × 224. Among them, some data pictures are from the CASIA-HWDB1.1 dataset, which is a publicly available HCCR dataset provided by the Institute of Automation, Chinese Academy of Sciences.

**Figure 5:**
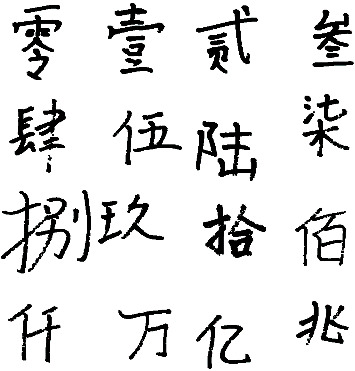
Examples of samples for each category in the dataset.

In addition, Figure 5 lists a sample of each category in the dataset. Different people's writing styles and even the habit of omitting or writing consecutively increase the diversity and richness of the dataset and improves the difficulty of identification.

### 4.2. Experimental setup

In this paper, the proportion of training and validation is 8 : 2. A total of three types of experiments are carried out, using two-way parallel, four-way parallel, and seven-way parallel ViT models, respectively. In addition, in each type of experiment, by changing the number of repeated stacks of encoders, we can know the relationship between network depth and model performance. In this paper, PyTorch is used to implement the network algorithm flow. The input original image size is 224 × 224, and the image is divided into 16 × 16 image blocks, so 196 image blocks can be obtained. When training the data set, 8 pictures are processed in each batch. The number of training times is set to 300, the learning rate is set to 0.003, the embedding dimension is 768, and the feed-forward subnetwork size is 3072. In addition, the stochastic gradient descent SGD optimization algorithm was used in the experiment to optimize its model. Dropout regularization is also used in the training process. By temporarily discarding some neuron connections randomly during the training process, the purpose is to effectively avoid overfitting of the model during the training process. At the same time, the generalization ability of the model is enhanced.

All experiments at this time are performed on a computer equipped with an Intel (R) Core (TM) i7-970 processor, 2 × 8 GB RAM, and a GeForce RTX 2060 graphics card with 6 GB of video memory.

### 4.3. Experimental Analysis and Discussion

This article has carried out three sets of experiments, which are two-way ViT, four-way ViT, and seven-way ViT. There is no method of pretraining on large datasets and then migrating to our dataset for fine-tuning training, but training directly on our dataset. Also, in all experiments, except for the number of encoder stacks, the other parameter settings are the same. In addition, the number of heads in the multihead attention is the same as the number of repetitions of each encoder. Among them, the number of training times is set to 300 times, and the learning rate is 0.003. Due to the different number of encoders, the final recognition accuracy and the parameter number of the model are also different.

All experimental results are clearly described in Table 4. When using the two-channel parallel ViT model training dataset, the number of encoders per channel is set to 6 layers. After the training is completed, the verification accuracy rate reaches 98.6%, and the parameter amount is 85.62 million. The FLOPs are 8.52 G. When using the four-channel parallel ViT model training dataset, by setting the number of encoders for each channel to 3 layers, the verification accuracy reaches 97.3%. The parameter number and the FLOPs are 85.62 million and 4.36 G, respectively. When using the seven-channel parallel ViT model training dataset, the number of encoders per channel is set to 3 layers. The verification accuracy is 97.1%. The FLOPs are 4.43 G. However, the parameter number is 148.38 million.

As we all know, the FLOPs required to process each picture are closely related to the number of network layers and the parameter amounts. Comparing the two-way parallel model and the four-way parallel model, when the parameter number and the total number of encoder layers are the same, the FLOPs of the four-way model are nearly half of the two-way model. Therefore, the parallel model can increase the speed of image processing. However, the parallel model method cannot improve the verification accuracy of the model.

**Figure 6:**
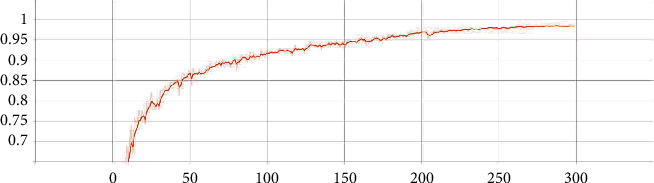
The visualization of the validation accuracy of the T-ViT model (3 encoders per channel).

The parameters and FLOPs of the best T-ViT model proposed in the paper and other models are shown in Table 5. When the number of encoders in each parallel channel of the proposed model T-ViT is 3, the model parameters and FLOPs are 43.11 million and 4.32 G, respectively. Compared with the other three models, T-ViT has the least number of parameters and FLOPs. Moreover, the visualization of the validation accuracy of the T-ViT model (3 encoders per channel) is shown in Figure 6. The abscissa represents epochs, and the ordinate represents the accuracy.

## 5. Conclusions and Future Work

This paper proposes a parallel and fast ViT method for offline HCCR, which divides the original picture into image blocks of the same size. Then the image blocks are flattened and linearly processed to form a vector sequence, which is encoded by the encoder, and finally, the category label is output by the MLP classification header. Different model structures of two-way parallel, four-way parallel, and seven-way parallel ViT were used to conduct comparative experiments on the handwritten Chinese character dataset. The experimental results prove the rationality and correctness of the model and show that the network can improve the accuracy of HCCR. In addition, this method not only has the advantage of being able to capture the interdependence between image sequence blocks through MHSA but also can effectively increase the speed of image recognition due to the parallelization of the encoder.

Last but not least, the model has certain limitations. First, the model needs to be trained on a dataset with a large amount of data as much as possible, which can ensure the final recognition accuracy and improve the generalization ability of the model. On the contrary, too many data sets will have a disadvantage, which is that it will cause the model to converge slowly. The dataset in this paper was made by randomly looking for volunteers, and the number is relatively small. However, a data expansion strategy is used in the early stages of the experiment to increase the number of datasets. Second, the higher the pixel value of the image in the dataset, the better the recognition accuracy. The model can be optimized in future research to achieve satisfactory accuracy on low-pixel image datasets. Finally, for the further development direction, it may focus on the use of knowledge distillation or model compression to reduce model parameters and combine them with other advanced models.

## Figures and Tables

**Table 1:** A summary table of offline HCCR related work.

Algorithm name	Brief methodology	Highlights	Limitations
MCDNN [30]	The model trained eight networks using different datasets, each with four convolutional layers and two fully connected layers.	It is the first model to successfully apply CNN to handwritten Chinese character recognition.	

R–CNN and ATR-CNN [31]	R-CNN consists of relaxation convolution layers whose neurons within a feature map do not share the same convolutional kernel. ATR-CNN further adopts an alternate training strategy, i.e., the weight parameters of a certain layer do not change by the backpropagation algorithm given a training epoch.	Relaxation convolution can be considered to enhance the learning ability of the neural network.	The replacement of the traditional convolutional layer with a relaxation convolution layer cannot further improve the recognition accuracy.

BP-NN [32]	The algorithm is improved by the selection of initial weights, excitation function, error function, and so on.	The method improves the speed and accuracy of offline handwritten Chinese character recognition.	The convergence speed is too slow, and it is easy to fall into the local minimum point.

HCCR-IncBN [33]	This model takes advantage of the sparse connections of the Inception module, performs convolution operations on the same input feature map at multiple scales, and uses 1 × 1 convolution kernels to compress data multiple times, which can increase the depth of the network and ensure that the computing resources are reduced.	The model has fewer training parameters, converges faster, and only requires 26 MB of storage space to store the entire model.	The recognition accuracy of the model is low.

SqueezeNet [34]	The proposed model retains small convolution kernels instead of large ones. In addition, the feature fusion algorithm between layers and the softmax function with L2-norm constraints are used.	The model parameters become less, the training becomes faster, and the portability is strong.	The accuracy of the model drops.

**Table 2:** A summary table of vision transformer related work.

Algorithm name	Brief methodology	Highlights	Limitations
CrossViT [35]	The architecture consists of a stack of K multiscale transformer encoders. Each multiscale transformer encoder uses two different branches to process image tokens of different sizes and fuses the tokens at the end with an efficient module based on cross-attention of the CLS tokens.	The dual-branch transformer combines image patches (i.e., tokens in a transformer) of different sizes to produce stronger image features.	The model increases in FLOPs and model parameters.

ViT and GCN [36]	Firstly, the scene image is divided into patches, and the positional encoding and vision transformer are used to encode the patches. Consequently, the long-range dependencies can be mined. On the other hand, the scene image is converted into superpixels.	Computing efficiency has been significantly improved.	The dataset is complex. The model is designed for higher-resolution vision tasks.

ViT [1]	First, the images under analysis are divided into patches, then converted into sequences by flattening and embedding. To maintain information about the position, the embedding position is added to these patches. Then, the resulting sequence is fed to several multihead attention layers to generate the final representation.	To boost the classification performance, the authors explore several data augmentation strategies to generate additional data for training.	The number of model parameters is large.

Vision transformer [37]	In this study, for the first time, authors utilized ViT to classify breast US images using different augmentation strategies. The results are provided as classification accuracy and area under the curve (AUC) metrics.	The results indicate that the ViT models have comparable efficiency with or even better than the CNNs in the classification of US breast images.	The authors use the strategy of transferring pretrained ViT models for further adaptation.

Convolutional vision transformer (CvT) [38]	This is accomplished through two primary modifications: a hierarchy of transformers containing a new convolutional token embedding and a convolutional transformer block leveraging a convolutional projection. These changes introduce desirable properties of convolutional neural networks (CNNs) to the ViT architecture.	The model has fewer parameters and lower FLOPs.	

Re-attention [39]	The model can regenerate the attention maps to increase their diversity at different layers with negligible computation and memory cost. The proposed method makes it feasible to train deeper ViT models with consistent performance improvements via minor modifications to existing ViT models.	The model has minimal computational and memory overhead.	

**Table 3:** Dataset characteristics.

Dataset	DHWDB
Number of class	16
Number of images per class	1810∼2575
Image size	224 × 224
Total number of images in the dataset	36210

**Table 4:** Performance of different models on the DHWDB dataset: parameters; FLOPs; accuracy.

Methods	Number of encoder layers per channel	Epochs	#Params (M)	FLOPs (G)	Acc. (%)
T-ViT	3	300	43.11	4.32	98.1
	4	300	57.28	5.72	98.3
	6	300	85.62	8.52	98.6

F-ViT	2	300	57.28	2.94	96.6
	3	300	85.62	4.36	97.3
	6	300	170.63	8.61	97.7

S-ViT	2	300	99.79	2.99	96.3
	3	300	148.38	4.43	97.1
	4	300	198.98	5.86	97.0

**Table 5:** The parameters and FLOPs of the best T-ViT model proposed and other models.

Model	#Params (M)	FLOPs (G)
ResNet-101 [46]	44.7	7.9
Swin-B [47]	88	15.4
CrossViT-18 [35]	43.3	9.03
T-ViT	43.11	4.32

## Data Availability

The all chart data used to support the findings of this study are included within the article.
